# Cardiac pacemaker channel (HCN4) inhibition and atrial arrhythmogenesis after releasing cardiac sympathetic activation

**DOI:** 10.1038/s41598-018-26099-9

**Published:** 2018-05-17

**Authors:** Kristine Chobanyan-Jürgens, Karsten Heusser, David Duncker, Christian Veltmann, Marcus May, Heidrun Mehling, Friedrich C. Luft, Christoph Schröder, Jens Jordan, Jens Tank

**Affiliations:** 1 Institute of Clinical Pharmacology, Hannover Medical School, Hannover, Germany; 2 Institute of Aerospace Medicine, German Aerospace Center, Cologne, Germany; 3 Department of Cardiology and Angiology, Hannover Medical School, Hannover, Germany; 4 Clinical Research Center Hannover, Hannover Medical School, Hannover, Germany; 5 Experimental Clinical Research Center, Charité Medical Faculty and Max Delbrück Center for Molecular Medicine, Berlin, Germany

## Abstract

Clinical trials and studies with ivabradine implicate cardiac pacemaker channels (HCN4) in the pathogenesis of atrial arrhythmias. Because acute changes in cardiac autonomic tone predispose to atrial arrhythmias, we studied humans in whom profound cardiac sympathetic activation was rapidly relieved to test influences of HCN4 inhibition with ivabradine on atrial arrhythmias. We tested 19 healthy participants with ivabradine, metoprolol, or placebo in a double blind, randomized, cross-over fashion on top of selective norepinephrine reuptake inhibition with reboxetine. Subjects underwent combined head up tilt plus lower body negative pressure testing followed by rapid return to the supine position. In the current secondary analysis with predefined endpoints before data unblinding, continuous finger blood pressure and ECG recordings were analyzed by two experienced cardiac electrophysiologists and a physician, blinded for treatment assignment. The total atrial premature activity (referred to as atrial events) at baseline did not differ between treatments. After backwards tilting, atrial events were significantly higher with ivabradine compared with metoprolol or with placebo. Unlike beta-adrenoreceptor blockade, HCN4 inhibition while lowering heart rate does not protect from atrial arrhythmias under conditions of experimental cardiac sympathetic activation. The model in addition to providing insight in the role of HCN4 in human atrial arrhythmogenesis may have utility in gauging potential atrial pro-arrhythmic drug properties.

## Introduction

Sympathetic and parasympathetic nervous system mechanisms are implicated in atrial arrhythmias. While parasympathetic activation promotes atrial arrhythmias in younger healthy individuals, adrenergic mechanisms may prevail in older individuals with cardiovascular disease^1^. Rapid fluctuations in cardiac autonomic tone potently promote atrial arrhythmias. Patients with focal ectopy originating from pulmonary veins exhibited transition from cardiac sympathetic to parasympathetic predominance just before paroxysmal atrial fibrillation onset^2^. Similarly, transitory cholinergic stimulations on top of pharmacological beta-adrenoreceptor stimulation elicited atrial tachyarrhythmia in isolated canine atrial preparations^3^. In contrast, vagal withdrawal preceded atrial fibrillation following coronary artery bypass surgery^4^. We established a human model of rapid cardiac vagal activation during profound cardiac sympathetic activation. Cardiac sympathetic activation is achieved through combined selective norepinephrine transporter inhibition and head-up tilt testing with lower body negative pressure. Rapid tilting back to the supine position acutely augments cardiac vagal activity while attenuating sympathetic drive. We applied the model in a placebo controlled, double blind, crossover study to test the hypothesis that hyperpolarization-activated and cyclic nucleotide-gated 4 (HCN4) channel inhibition with ivabradine promotes atrial arrhythmogenesis in healthy individuals. In clinical trials, atrial fibrillation was more likely to occur in ivabradine compared with placebo treated patients^5,6^. Moreover, trafficking-defective mutations in HCN4 gene predispose to early-onset atrial fibrillation^7^. Beta-adrenoreceptor blockade, which in addition to lowering heart rate attenuates adrenergic influences on atrial and ventricular myocardial cells, served as control intervention.

## Results

**Figure 1:**
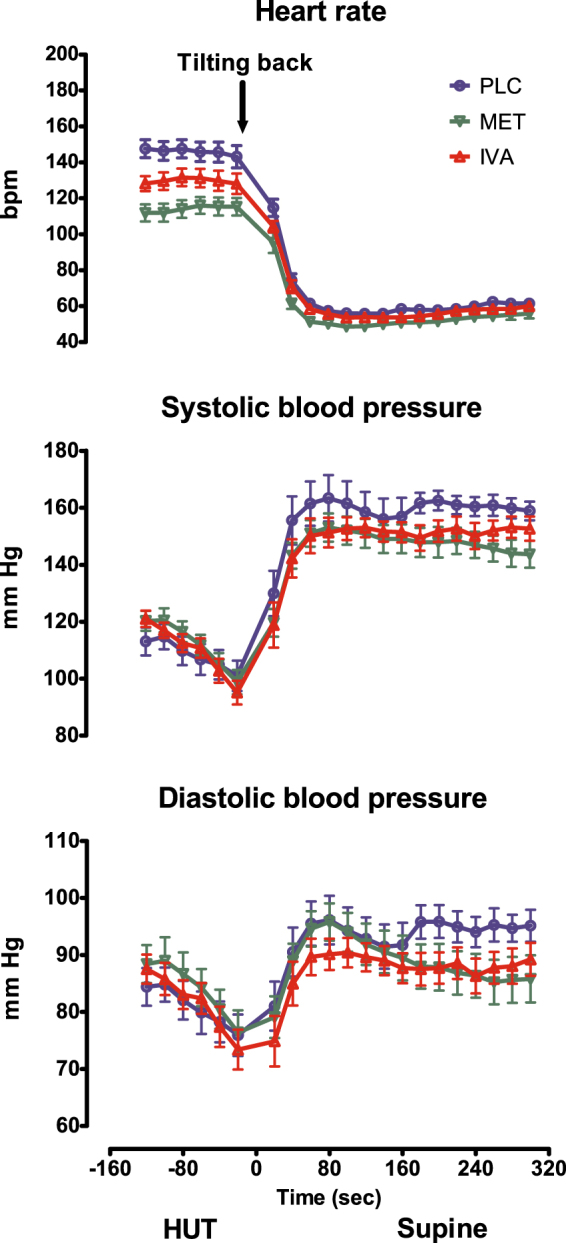
Hemodynamics during transition from head-up tilt (HUT) to the supine position. Ivabradine (IVA), metoprolol (MET) and placebo (PLC).

Heart rate and blood pressure responses at the end of the head-up tilt protocol and the transition to the supine recovery phase are shown in Fig. 1. Combination of norepinephrine transporter inhibition and severe orthostatic stress resulted in substantial tachycardia that was attenuated by, both, metoprolol, and ivabradine. Return to the supine position led to rapid heart rate and blood pressure recovery regardless of treatment.

**Figure 2:**
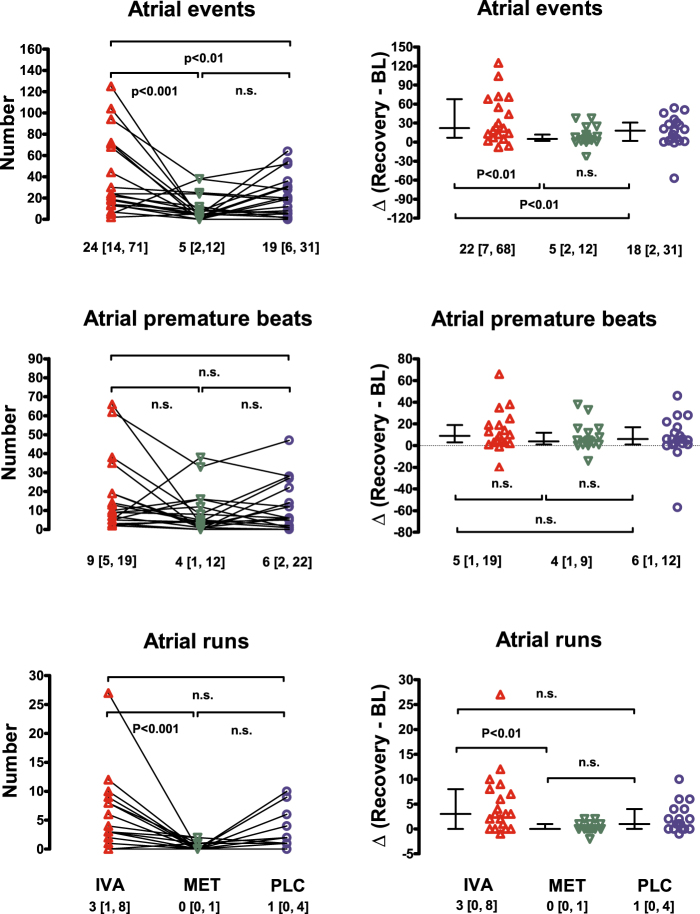
Atrial events, atrial premature beats and atrial runs after tilting back (recovery) as individual scatter plots (left panels) and after correction to baseline (BL) with ivabradine (IVA), metoprolol (MET) and placebo (PLC) (right panels).

During the five minutes recovery phase following orthostatic testing, atrial events were significantly more frequent and more pronounced with ivabradine compared with metoprolol or with placebo (Fig. 2). Six participants out of 19 showed a particularly prominent response to ivabradine. Of those, three were allocated to the treatment sequence metoprolol-placebo-ivabradine, two to ivabradine-placebo-metoprolol, and one to placebo-ivabradine-metoprolol. Thus, the arrhythmogenic effect of ivabradine was not restricted to one particular treatment sequence. Yet, a significant sequence effect cannot be excluded given the low number of participants in each treatment sequence. Two participants showed numerically higher atrial events on placebo day than on ivabradine. One participant developed more atrial events on metoprolol than on placebo and ivabradine.

**Figure 3:**
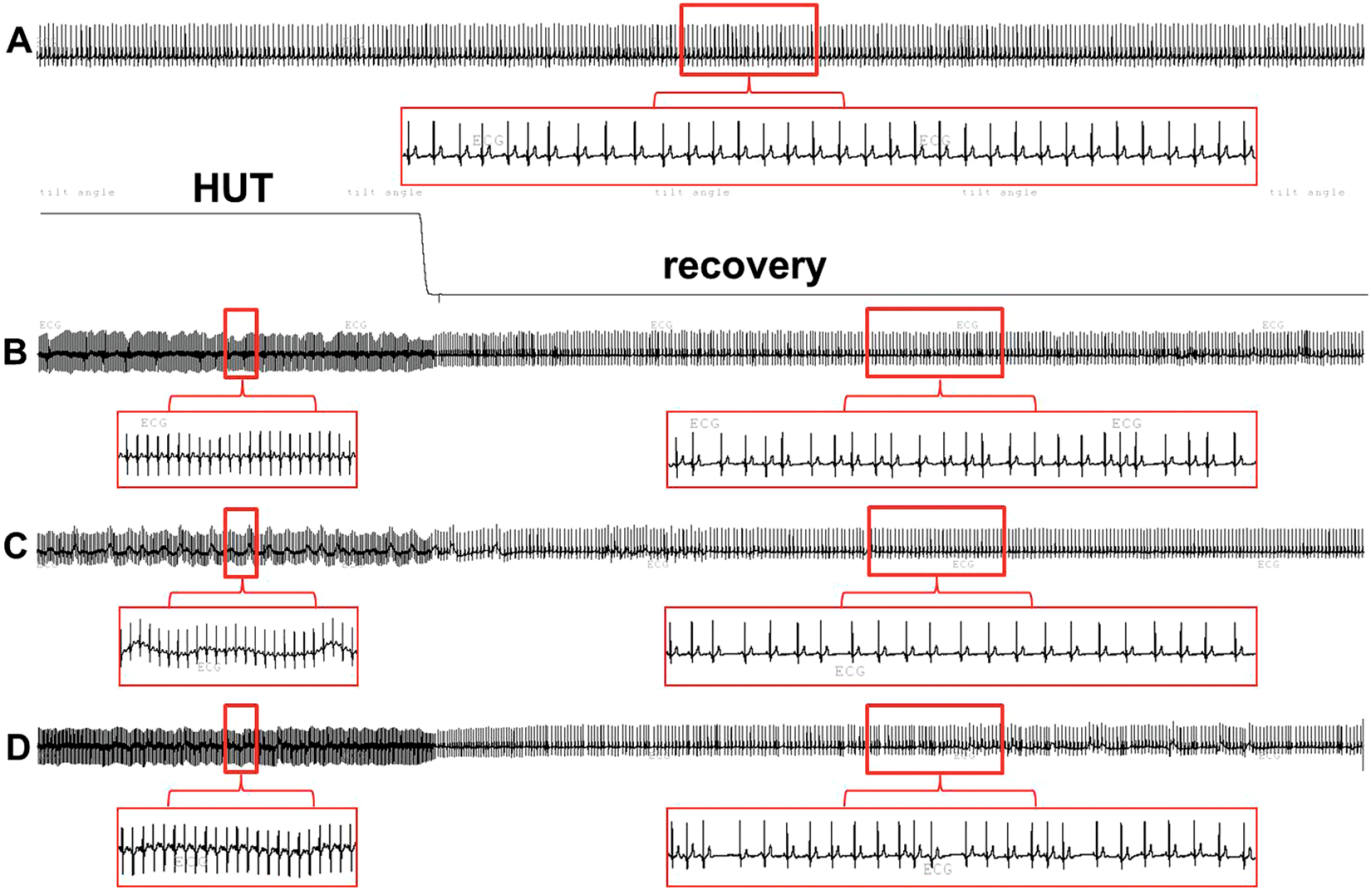
ECG recordings of the participant who experienced the highest number of atrial premature beats after tilting back in all three treatment occasions: (**A**) in the baseline supine position on the ivabradine day; (**B**–**D**) tilting back from head-up tilt into supine position on ivabradine (**B**), metoprolol (**C**), and placebo (**D**) days.

When atrial arrhythmias were observed separately as atrial premature beats and atrial runs, statistically significant difference was seen in the number of atrial runs between ivabradine and metoprolol treatment (P < 0.001; Fig. 2). Figure 3 shows ECG recordings during the recovery period on ivabradine, on metoprolol, and on placebo in a participant experiencing the highest number of atrial arrhythmias. The number of atrial premature beats was numerically higher with ivabradine compared to metoprolol and placebo (Table 1), the group difference was not statistically significant (P = 0.071 for all treatments). In contrast, we observed no clinically relevant or statistically significant differences in atrial or ventricular arrhythmia rates between treatments in the supine baseline period before orthostatic testing, although numerically more atrial events were counted on placebo and on ivabradine compared with metoprolol (Table 1). No differences in other arrhythmia types at baseline or after tilting back were observed. Table 1 summarizes the number of arrhythmic events in all three study occasions.

**Table 1:** The number of subjects with atrial events, atrial premature beats, atrial runs and ventricular premature beats on different study days at supine baseline and after tilting back (recovery) and the number of respective arrhythmic events per study occasion (in parenthesis).

Day	Atrial events		Atrial premature beats		Atrial runs		Ventricular premature beats	
	Baseline	Recovery	Baseline	Recovery	Baseline	Recovery	Baseline	Recovery
IVA	4 (98)	19 (770)	4 (75)	19 (326)	2 (5)	15 (101)	1 (2)	4 (82)
MET	1 (23)	16 (190)	1 (14)	16 (160)	1 (2)	5 (7)	0 (0)	1 (3)
PLC	6 (131)	17 (413)	6 (92)	17 (225)	3 (5)	13 (44)	0 (0)	3 (60)

IVA, ivabradine; MET, metoprolol; PLC, placebo.

## Discussion

The important finding of our study is that selective HCN4 inhibition with ivabradine increases atrial arrhythmic event rate compared with the beta-adrenoreceptor blocker metoprolol or with placebo during transition from profound cardiac sympathetic activation to sympathetic withdrawal and cardiac vagal activation. In addition to introducing a novel model for human drug research, our study may have implications for the clinical use of HCN4 inhibitors and provide insight in the role of this channel in the pathophysiology of human atrial arrhythmias.

Combined norepinephrine transporter inhibition and orthostatic stress produced profound cardiac sympathetic activation^8,9^. Sympathetic withdrawal and cardiac vagal activation was elicited by rapidly tilting subjects back to the supine position. In peripheral tissues, norepinephrine transporter inhibition tends to increase norepinephrine availability^10^. Conversely, norepinephrine transporter inhibition decreases sympathetic outflow from the brain^11,12^. Overall, sympathetic activity is redistributed towards the heart^13,14^. Therefore, orthostatic tachycardia is a hallmark of pharmacological norepinephrine transporter inhibition^9,15^ and familial norepinephrine transporter dysfunction^16^. Yet, baroreflex heart rate regulation is maintained such that baroreflex unloading with return to the supine position rapidly reduced heart rate^8,17^. Given the transmission characteristics of efferent vagal and sympathetic fibers, vagal activation may have preceded sympathetic withdrawal during this phase. While heart rate variability measurements provide additional insight in autonomic control mechanisms, we opted not to conduct this analysis given the large number of arrhythmic events and the non-steady-state conditions.

Our findings confirm and extend observations in genetic conditions associated with altered HCN4 function and clinical trials with the HCN4 blocker ivabradine^18,19^. Rare HCN4 gene mutations have been identified in patients with familial bradycardia and atrial fibrillation^19,20^. Trafficking-defective, loss of function mutations in the HCN4 gene predispose to early-onset atrial fibrillation in individuals with healthy hearts^7^. Furthermore, ivabradine treatment predisposed to atrial fibrillation compared with placebo treated patients^5,6^. Conversely, ivabradine on top of beta-adrenoreceptor blockade appeared to be beneficial in patients with atrial fibrillation in smaller studies^21,22^. Our study suggests that HCN4’s full atrial arrhythmogenic potential may be revealed during transition from cardiac sympathetic activation to vagal predominance.

Both autonomic nervous system branches affect cardiac arrhythmogenicity in a complex fashion^1–4^. For example, vagotonic maneuvers trigger atrial arrhythmias in susceptible individuals^23,24^. Influences of cardiovascular medications on cardiac arrhythmias during changes of cardiac autonomic tone may go undetected during routine clinical development, particularly in early stage clinical trials in healthy persons.

Differential effects of metoprolol and ivabradine on atrial arrhythmogenicity likely result from drug specific interactions with cardiac autonomic drive and influences on endogenous rhythm generation. Beta-adrenoreceptor blockade attenuates sympathetic influences on sinus node, conduction system, and atrial and ventricular cardiomyocytes. Ivabradine, which binds to the open HCN4 channel^25,26^, selectively slows sinus node diastolic depolarization while leaving sympathetic activation elsewhere in the heart unopposed^8,17^. Aggravated bradycardia during baroreflex loading suggested that ivabradine might also augment vagal influences on the sinus node^17^. Furthermore, HCN4 appears to serve as defense mechanism against bradycardia and to stabilize cardiac rhythm^27^.

Overall, we propose that ivabradine-induced arrhythmogenesis may be explained at least in part by unopposed cardiac sympathetic activation obscured by sinus rate reduction. In addition, we speculate that HCN4 inhibition might perturb the fine interplay and synchronization between membrane and calcium clocks involved in cardiac pacemaking^28–30^.

A potential limitation of our study is that for practical reasons, particularly ethically acceptable treatment duration in healthy persons, test drugs were not in steady state. Yet, we previously showed that we achieved drug concentrations in a clinically relevant range^8^. Nevertheless, our findings should not be simply extrapolated to patients chronically treated with these drugs. Furthermore, we retrospectively analyzed data collected during a clinical trial. We attempted to minimize potential sources of bias by prospectively defining study endpoints as well as the statistical analysis plan before data was compiled and analyzed and by blinding investigators rating ECG tracings.

We conclude that HCN4 inhibition with ivabradine, compared with beta-adrenoreceptor blocker metoprolol, while lowering heart rate did not protect from atrial arrhythmias during transition from cardiac sympathetic activation to vagal predominance. Our approach of combining pharmacological and physiological methodologies eliciting rapid and profound changes in cardiac autonomic tone may have utility in testing the atrial arrhythmogenicity of drugs. In patients with heart failure or angina pectoris, ivabradine is commonly prescribed on top of beta-adrenoreceptor blockade. The clinical implication of our study is that patients treated with ivabradine without beta-adrenoreceptor blockade may require intensified monitoring for atrial arrhythmias. Additionally, beta-adrenoreceptor blockers may be necessary to prevent ivabradine-induced cardiac rhythm derangements in certain patients with high risk for or already overt atrial arrhythmia. The issue may be particularly relevant for patients with postural tachycardia syndrome who feature orthostatic heart rate responses with standing that are almost identical to those observed on norepinephrine transporter inhibition in our study.

## Methods

### Study participants

We conducted an observer-blinded, prospectively planned, secondary analysis of data previously obtained in a mechanism-oriented clinical investigation (clinicaltrials.gov: NCT00865917)^8^. The study was performed in accordance with the Declaration of Helsinki and approved by the national competent authority and local institutional review boards (the ethics committee of Charité – Universitätsmedizin Berlin as well as the ethics committee of Hannover Medical School). Nineteen healthy normotensive men (18–40 years, BMI 18–30 kg/m^2^, resting heart rate >55 bpm) were included after written informed consent had been obtained.

### Protocol

The protocol has been described previously^8^. Briefly, in a randomized, double-blind, three-period, six-sequence crossover fashion, subjects ingested maximal recommended doses of metoprolol (95 mg), ivabradine (7.5 mg), or placebo 13 h and 1 h before testing on three separate study days. In addition, participants ingested 4 mg of selective norepinephrine transporter inhibitor reboxetine (Edronax, Pfizer, Karlsruhe, Germany) 13 h and 1 h before testing as background medication on all three study days. The washout period between measurements was at least 2 weeks to prevent carry over effects.

Cardiovascular testing was conducted between 08:00 and 11:00 a.m. in a quiet laboratory at an ambient temperature of 22–23 °C. Heart rate was continuously monitored by electrocardiogram (ECG, Viridia, Hewlett-Packard). Beat-to-beat finger blood pressure was continuously registered by volume-clamp photoplethysmography (2300 Finapres, Ohmeda, Madison, WI) with the finger kept at the heart level throughout the experiment. After instrumentation, subjects remained supine for 30 min for baseline recordings. Head-up tilt was started with a graded initial phase during which the tilt angle was increased by 15° every 2 min up to a tilt angle of 60°, at which subjects remained for 20 min. With the subjects remaining at 60° head-up tilt, additional orthostatic stress was applied stepwise using lower body negative pressure of −20 and −40 mm Hg for 10 minutes each. The complete head-up tilt protocol lasted up to 46 minutes. The test was aborted when subjects experienced hypotension or presyncopal symptoms. At the end of the test, lower body negative pressure was switched off and subjects were rapidly returned to the supine position. Thereafter, recordings were continued during the recovery period for another five minutes.

### Data acquisition and analysis

ECG and continuous finger blood pressure signals were recorded at 500 Hz using the Windaq pro+ software (Dataq Instruments, Akron, OH). Five-minute ECG recordings during supine baseline and after tilting back were first analyzed by two experienced cardiac electrophysiologists (DD, CV). Discrepancies were resolved through discussion with another physician experienced in ECG analysis (KCJ). Evaluators were blinded for patient and treatment. Premature atrial and ventricular beats were assessed. An atrial or ventricular run was defined as 4–7 successive premature beats. An ectopic rhythm was defined as a P wave showing a differing morphology and/or sudden jump in cycle length compared to P wave morphology or cycle length during sinus rhythm. Sinus arrest was present when regular ventricular rhythm without P waves was identified. An atrioventricular (AV) block was registered when AV dissociation was detected. Arrhythmias were counted per minute as well as for the entire five minutes interval. Different counts between evaluators were reconciled by averaging and rounding up. The total atrial premature activity was accounted as the sum of single premature atrial events and all events during an atrial run.

### Endpoint and statistical analysis

Exploratory endpoints were defined and the statistical analysis approach was finalized before the analysis was unblinded. Our exploratory endpoints include the total number of atrial arrhythmic premature events over five min observation period after tilting back (further referred as atrial events), the number of arrhythmic events during supine baseline, and the difference between the counts of arrhythmic events after tilting back corrected to the arrhythmic events during supine baseline (delta). D’Agostino and Pearson omnibus normality test was applied for data distribution testing. The non-parametric Friedman test comparing paired groups together with Dunn’s post hoc test for multiple comparisons were used to test the differences in the arrhythmic events rate between the treatments (ivabradine vs. metoprolol vs. placebo) as well as between visit days (visit 1 vs. visit 2 vs. visit 3, treatment-independent One-way analysis of variance (ANOVA)). Potential influence of sequence administration on atrial arrhythmogenicity was considered. P values < 0.05 were considered statistically significant. Data are presented as median [25^th^, 75^th^ percentile] (for arrhythmic events) and as mean ± SEM (for hemodynamic parameters), respectively. All analyses were performed using GraphPad Prism version 5.00 for Windows, GraphPad Software, San Diego, California, USA.

### Data Availability

The datasets generated during and/or analysed during the current study are available from the corresponding author on reasonable request.
